# Forecasting Stomach Cancer Burden from High Sodium Intake in Japan, 2022–2050: Scenario Analysis of Demographic Disparities

**DOI:** 10.3390/nu18101641

**Published:** 2026-05-21

**Authors:** Constanza De Matteu Monteiro, Daisuke Yoneoka, Shuhei Nomura

**Affiliations:** 1 National Food Institute, Technical University of Denmark, 2800 Kongens Lyngby, Denmark; cdmmo@food.dtu.dk; 2 National Institute of Infectious Disease, Japan Institute for Health Security, Tokyo 162-8640, Japan; yoneoka@niid.go.jp; 3 Department of Global Health Policy, Graduate School of Medicine, The University of Tokyo, Tokyo 113-0033, Japan; 4 Global Health Policy Lab, International Research Institute of Disaster Science (IRIDeS), Tohoku University, Sendai 980-8572, Japan; 5 Department of Health Policy and Management, School of Medicine, Keio University, Tokyo 160-8582, Japan; 6 Department of Food, Nutrition and Health, Graduate School of International University of Health and Welfare, Tokyo 107-8402, Japan

**Keywords:** stomach cancer, sodium intake, disability-adjusted life years, forecasting, Japan, Health Japan 21, Global Burden of Disease

## Abstract

Background/Objectives: High sodium intake is a leading dietary risk factor for stomach cancer, particularly in East Asia. In Japan, traditional dietary patterns contribute to elevated sodium consumption and a high burden of stomach cancer. This study aims to forecast disability-adjusted life years (DALYs) for stomach cancer attributable to high sodium intake in Japan from 2022 to 2050, and to assess the impact of multiple sodium reduction policy scenarios. Methods: We conducted a longitudinal forecasting study using autoregressive integrated moving average with exogenous variables (ARIMAX) models based on Global Burden of Disease 2021 data (1990–2021). The Japanese population was stratified by sex and age groups (15–49, 50–69, and ≥70). Five future exposure scenarios were modelled: (1) reference (current trends), (2) best-case (50% reduction in sodium exposure by 2050), (3) optimal (30% reduction by 2032), (4) moderate (30% reduction by 2050), and (5) worst-case (highest exposure levels from recent years maintained). These scenarios were aligned with national and international sodium reduction targets, including the revised “Health Japan 21” (third term; 7 g/day by 2032) and the World Health Organisation (WHO) 5 g/day/30% reduction goals. Results: Under the reference scenario, age-standardised DALY rates are projected to decline by 31.4% (to 15.4 per 100,000) by 2050. The best-case scenario projects a 54.7% decline (to 10.1 per 100,000). Substantial demographic disparities persist: males and those aged ≥70 consistently show higher burdens. Notably, the 50–69 age group shows the greatest variation in 2050 projections across scenarios (17.1 to 73.5 per 100,000), indicating high policy sensitivity. Meanwhile, in the ≥70 group, DALY rates remain high regardless of scenario, especially among males (199.4 vs. 57.8 per 100,000 for females), reflecting cumulative lifetime exposure. Conclusions: Under modelled assumptions, sustained achievement of national sodium reduction targets could meaningfully reduce future stomach cancer DALYs in Japan, with the largest absolute gains in older adults but the largest relative gains in younger and middle-aged groups. Because stomach cancer aetiology is multifactorial and the projections rest on modelled associations and a continuity-of-trend assumption, these findings support strengthened, demographically targeted sodium reduction interventions as one complementary component of a broader, multi-risk factor approach to stomach cancer prevention.

## 1. Introduction

Excess sodium intake remains a critical public health concern worldwide, with substantial evidence linking high sodium consumption to adverse health outcomes, particularly non-communicable diseases (NCDs) [1]. In oncology, the association between high sodium intake and stomach (gastric) cancer is supported by extensive epidemiological evidence, including multiple meta-analyses and a large international pooled analysis [2,3,4]. The Global Burden of Disease (GBD) 2021 study, consistent with earlier iterations, identified diets high in sodium as one of the leading dietary risk factors for stomach cancer, accounting for 7.9% of stomach-cancer-related disability-adjusted life years (DALYs) worldwide in 2021 [5]. To summarise the magnitude of these effects, the most recent comprehensive meta-analysis of 38 case-control studies (37,225 participants) reported a pooled odds ratio (OR) of 1.55 (95% CI 1.45–1.64; *p* < 0.001) for high versus low dietary salt intake [4]. Consistent with this, the Stomach cancer Pooling (StoP) Project individual-participant pooled analysis of 25 studies (10,283 cases and 24,643 controls) reported adjusted ORs of 1.59 (95% CI 1.25–2.03) for salty taste preference, 1.33 (95% CI 1.16–1.54) for habitual addition of table salt, and 1.24 (95% CI 1.01–1.51) for the highest versus lowest tertile of high-salt and salt-preserved food intake [3]. Risk estimates have been broadly consistent across geographical settings and study designs, although associations are typically stronger for direct markers of high-salt-eating behaviour (e.g., salty taste preference, addition of table salt, and salt-preserved foods) than for self-reported total sodium intake, reflecting a well-known measurement error in dietary sodium assessment [2,3,4].

The burden of stomach cancer shows marked geographical variation, with East Asian countries, including Japan, displaying particularly high incidence; East Asia alone accounted for 50.8% of new cases globally in 2021 [6]. Within East Asia, however, salt-attributable stomach cancer burden varies appreciably across countries: 2021 age-standardised salt-attributable DALY rates are 22.3, 41.5 and 24.1 per 100,000 in Japan, mainland China and South Korea, respectively, versus 20.8 globally [7]. This regional pattern reflects a combination of dietary habits characterised by high salt consumption (including traditional food preservation and seasonings such as miso and soy sauce), long-standing culinary preferences, and a historically high prevalence of *Helicobacter pylori* infection [8]. Mechanistically, high sodium damages the gastric mucosal barrier and induces parietal-cell loss and altered mucin production, predisposing the stomach to atrophic gastritis and intestinal metaplasia—recognised precursors of gastric adenocarcinoma [9,10]. These pathways act synergistically with *H. pylori* through the up-regulation of CagA, amplified pro-inflammatory signalling and increased endogenous DNA damage [9,11]. The salt–*H. pylori* synergy underpins our analytical framework: population-level sodium reduction in Japan, where *H. pylori* prevalence remains substantial in older birth cohorts [8], has potential to mitigate stomach cancer risk through both direct mucosal effects and attenuation of *H. pylori*-driven carcinogenesis. In Japan, stomach cancer remains a leading cause of cancer mortality [12], and GBD 2021 ranks high sodium intake as one of the most important dietary risk factors contributing to this burden [5]. Specifically, it ranks third in male cancer mortality (~11%, after lung and colorectal cancers) and fifth in female cancer mortality (~8%, after colorectal, lung, pancreatic and breast cancers) [13]. Stomach cancer in Japan also displays a marked male preponderance: a 2019 male-to-female incidence ratio of ~2.2:1 and a 2021 salt-attributable DALY rate ratio of ~2.5:1 (33.1 vs. 13.0 per 100,000) [7,13]. This sex disparity reflects differential prevalence of risk factors (smoking, alcohol, and historically higher male *H. pylori* prevalence), greater male intake of salt-rich foods, and oestrogen-mediated mucosal protection that diminishes after menopause [14]. These sex differences motivate the demographic stratification adopted in the present forecasts.

Several previous modelling studies have used comparative risk assessment or life-table approaches to examine the health impact of sodium reduction. Studies in Cameroon, Singapore and Australia projected the effect of population-level sodium reduction strategies (e.g., achieving the World Health Organisation’s [WHO] 30% reduction target or mandating packaged-food sodium benchmarks) primarily on the cardiovascular disease burden in those settings [15,16,17], and a global 2016–2040 forecasting study using GBD 2016 data projected future mortality across 250 causes of death in 195 countries [18]. For Asian settings, Ning et al. used GBD 2019 to describe the 1990–2019 trend in gastric cancer across five Asian countries, including Japan, but did not forecast future burden or model policy scenarios [19]; Takachi et al. estimated the 2015 cross-sectional cancer burden attributable to highly salted food in Japan without projecting future trajectories [20]. Most directly related to the present work, our group has previously forecast DALYs for cardiovascular disease, chronic kidney disease and stomach cancer attributable to salt intake in Japan for 2017–2040, using GBD 2017 and scenarios anchored to the second term of the “Health Japan 21” national health promotion policy (daily salt target of 8 g/day) [21].

Despite this evidence base, three gaps remain that are of direct relevance to current Japanese health policy. First, the Japanese national salt reduction target was revised in 2024 with the launch of the third term of “Health Japan 21”, which lowered the adult daily salt intake target from 8 g/day (2022) to 7 g/day (2032) [22,23]; no existing forecast explicitly aligns projected DALYs with this revised target. Second, GBD 2021 incorporated substantial methodological updates—including the Burden-of-Proof framework for quantifying the evidence of risk [24] and updated estimates of exposure–response relationships for dietary sodium [5]—that had not been reflected in earlier stomach-cancer-focused Japanese projections. Third, National Health and Nutrition Survey (NHNS) data indicate that the long-standing decline in population salt intake stalled after 2010 and that intake rose in several age groups during 2015–2021 [25], a pattern that has not been propagated into prior forecasts of attributable burden.

To address these gaps, we forecast DALYs for stomach cancer attributable to diets high in sodium in Japan from 2022 to 2050 using autoregressive integrated moving average models with exogenous variables (ARIMAX) models fitted to GBD 2021 data, and we compare five future exposure scenarios aligned with (i) the revised “Health Japan 21” (third term) target of 7 g/day by 2032, (ii) the WHO 30% reduction/5 g/day targets, (iii) a reference scenario extrapolating observed trends, and (iv) a worst-case scenario reflecting the recent NHNS-observed stalling of progress. Projections are produced at both the age-standardised level and by joint sex × age strata (15–49, 50–69, ≥70 years). To our knowledge, this is the first study to (a) benchmark Japanese stomach cancer DALY forecasts against the revised third-term Health Japan 21 target using GBD 2021, (b) integrate the post-2015 stalling of sodium intake reduction into long-range projections, and (c) quantify demographic disparities in projected burden under each scenario. We hypothesised that achieving the revised national target would reduce future DALY rates substantially but would fall short of WHO-aligned targets, and that projected relative and absolute disparities by sex and age would persist or widen under all but the most aggressive scenarios.

## 2. Materials and Methods

This longitudinal forecasting study used a two-stage modelling approach to project the burden of stomach cancer attributable to high sodium intake in Japan from 2022 to 2050. We developed multiple future exposure scenarios based on national and international sodium reduction targets. The overall forecasting model structure follows methodologies previously reported elsewhere [18,21].

### 2.1. Attributable DALYs and Predictor Variables: 1990–2021

We extracted data from the GBD Results tool for the years 1990–2021 for Japan [26]. Age-standardised DALY rates (per 100,000 population) for stomach cancer attributable to diets high in sodium and the summary exposure value (SEV) for diets high in sodium were extracted by sex (females, males and sexes combined) and in age-stratified form for adults aged 15–49, 50–69 and ≥70 years. We also retrieved the socio-demographic index (SDI)—a composite measure (scaled 0–1) of sociodemographic development based on fertility rate in adults under 25 years, education level and income per capita [5]—for all ages and sexes combined as an additional predictor variable.

### 2.2. Future Exposure Scenarios

We constructed four alternative future exposure scenarios in addition to a reference forecast. The reference forecast assumes that current trends observed from historical data from 1990–2021 are maintained. The alternative scenarios (S1–S4) were defined as follows:

Best-case scenario (S1): targets a 50% reduction in SEV for diets high in sodium by 2050, modelled as a constant monotonic decreasing function from 2022 to 2050. Given that the average adult salt intake in Japan was approximately 9.8 g/day in 2023 [25], this 50% reduction would align closely with the WHO recommendation of less than 5 g/day [1]. Optimal scenario (S2) and moderate scenario (S3): both target a 30% reduction in SEV for diets high in sodium, reflecting goals similar to the revised “Health Japan 21” (third term) target of up to 7 g/day at the population level [22]. Under S2, the 30% reduction is achieved by 2032 and then maintained to 2050; under S3, the 30% reduction is achieved linearly by 2050. Worse-case scenario (S4): assumes that the highest exposure of dietary sodium observed during the last five years is maintained as a constant until 2050. To validate our assumptions about SEV reduction rates relative to salt intake recommendations, we examined the relationship between salt intake and SEV using data from a previous study in Japan [21].

### 2.3. Statistical Analysis and Forecasting Models

We employed the ARIMA model for forecasting values from 2022 to 2050, building on established methodology from previous studies [18,21,27,28]. The autoregressive (AR) term estimates the influence of past observations on future values; the moving average (MA) term incorporates previous (white noise) errors over time; and the integrated (I) term addresses non-stationarity in the time series by calculating differences between consecutive observations until the series achieves stationarity. The model is specified by three parameters, denoted ARIMA (p, d, q), where p refers to the order of the AR term, d is the number of differencing steps in the I term, and q is the order of the MA term.

Before model fitting, we assessed time-series stationarity using the Dickey–Fuller test. Autocorrelation and partial autocorrelation functions were used to determine stationarity. Model parameters were estimated using maximum likelihood, with the optimal orders (p, d, q) selected based on the Akaike Information Criterion (AIC).

Our prediction followed a two-step procedure. First, we independently predicted the predictor variables (SEV for diets high in sodium and SDI). Second, log-transformed attributable DALY rates were modelled, incorporating the predicted values from step 1 prediction using a vanilla ARIMA model with exogenous variables (ARIMAX), following the methodology detailed by Nomura et al. (2020) [21]. Forecasts were generated under each scenario together with 95% prediction intervals derived from the asymptotic normal distribution. Models were developed for each age and sex category. Two-tailed tests were used throughout, with statistical significance set at *p* < 0.05. All analyses were conducted in R version 4.2.2, using the “forecast” and “tseries” packages.

## 3. Results

### 3.1. Overall Trends in Disease Burden and Risk Exposure

From 1990 to 2021, Japan experienced a substantial decline in the age-standardised DALY rate for stomach cancer attributable to diets high in sodium, concurrent with an increase in SDI (Table 1). The burden consistently showed marked sex and age disparities, with males and older age groups experiencing higher attributable DALYs across all time periods (Supplementary Table S1).

The exposure to dietary sodium, measured by SEV, showed distinct temporal patterns. The period from 2000 to 2010 saw a substantial decrease in dietary sodium exposure (11.8% in SEV for both sexes combined; −10.9% for males and −12.7% for females), while the subsequent decade showed only minimal reduction (<3% in SEV across all sex categories) (Table 1). Analysis of more recent trends revealed concerning patterns: data from the past five years indicated an increasing trend in dietary sodium exposure, with one notable exception: adults aged 50–69 years (both sexes) showed a consistent decreasing trend since 2017 (Supplementary Figure S1). Consequently, for all other age categories, including age-standardised analyses, the 2021 SEV values—representing the highest SEV in the recent five-year period—were used to construct the worst-case scenario (S4). Based on the 2016 sex-combined data, our analysis showed that an SEV of approximately 39.2% corresponded to a daily salt intake of 9.9 g (Supplementary Figure S2).

### 3.2. Forecasted Trends in Disease Burden

**Figure 1:**
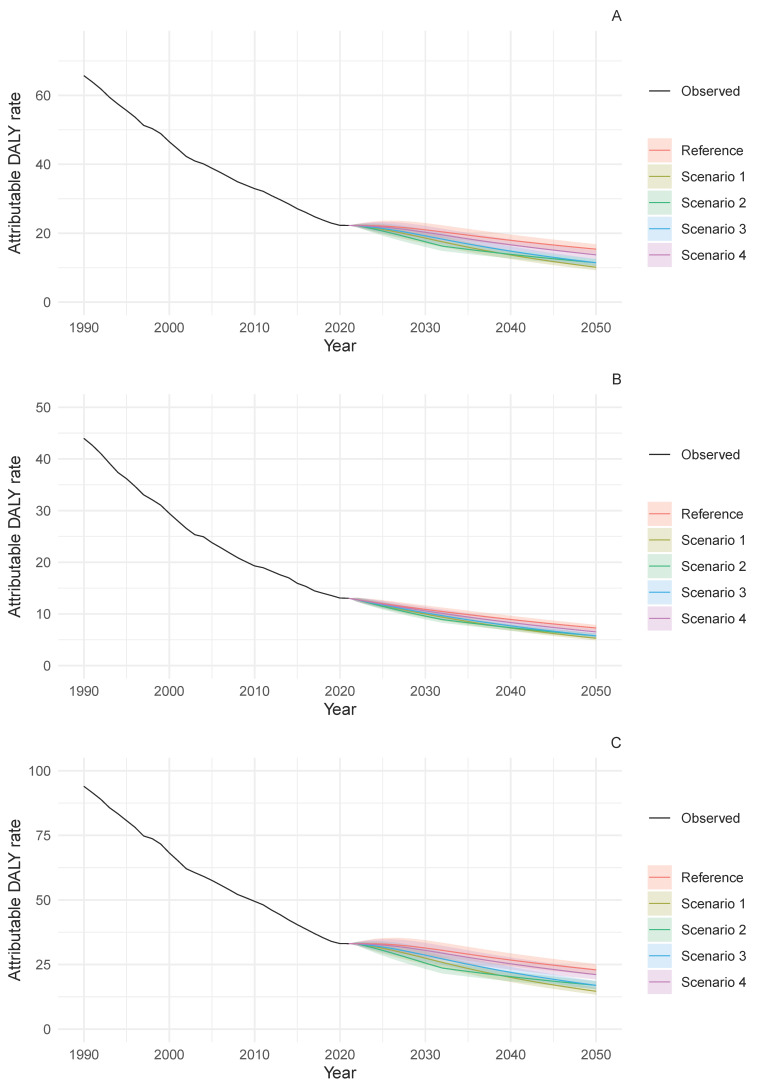
Trends in age-standardised DALY rates attributable to diets high in sodium for stomach cancer in Japan, 1990–2050. (**A**) Sexes combined, (**B**) females and (**C**) males. DALY rates are per 100,000 population. Lines show observed data (1990–2021) and projections (2022–2050) with 95% prediction intervals. Reference scenario extrapolates historical trends. Alternative scenarios: best-case scenario (S1) targets 50% SEV reduction; optimal scenario (S2) and moderate scenario (S3) target 30% SEV reduction by 2032 and 2050, respectively; worst-case scenario (S4) assumes that the highest dietary-sodium exposure observed in the last five years is maintained. SEV = summary exposure value.

Figure 1 presents the age-standardised DALY rates attributable to diets high in sodium for stomach cancer, showing both historical data (1990–2021) and forecasted trends under reference and alternative future scenarios, stratified by sex. All scenarios demonstrated a continuing decline in attributable DALY rates, albeit at varying magnitudes and with marked differences between intervention pathways.

By 2050, the best-case scenario (S1), which targeted a 50% reduction in SEV, projected the lowest attributable DALY rates per 100,000 population: 14.6 (95% prediction interval [PI] 13.3–16.1) for males, 5.3 (95% PI 4.9–5.7) for females, and 10.1 (95% PI 9.3–11.1) for the combined population. These projections represent substantial reductions from 2021 levels of 33.1 for males (55.9% reduction), 13.0 for females (59.2% reduction), and 22.3 for the combined population (54.7% reduction). Under the reference scenario, which extrapolates current trends, rates were projected to decrease to 22.9 (95% PI 20.9–25.2) for males (30.8% reduction from 2021), 7.3 (95% PI 6.7–7.9) for females (43.8% reduction), and 15.4 (95% PI 14.1–16.8) for the combined population (31.4% reduction). The optimal (S2) and moderate (S3) scenarios, both targeting a 30% reduction in SEV but with different implementation timelines, yielded projected rates of 16.9 (95% PI 15.4–18.6) for males, 5.8 (95% PI 5.3–6.2) for females, and 11.4 (95% PI 10.5–12.5) for the combined population—representing reductions of 48.9%, 55.4%, and 48.9% from 2021 levels, respectively. Scenarios S2 and S3 exhibited distinct trajectories regarding the timing of targeted reductions in dietary sodium exposure: S2, which simulated reductions at a faster pace, showed comparable performance to the best-case scenario (S1) until 2039, at which point S1 began to demonstrate superior outcomes in mean predicted values (Supplementary Table S2).

Owing to the observed trend of increased SEV for diets high in sodium in recent years, the reference scenario is forecast to be either worse (based on mean predicted values) or very similar (considering the 95% PI) to S4 (worst case). The reference scenario showed an additional burden of approximately 1–2 age-standardised DALYs per 100,000 population across all sex categories compared with S4. Compared with the reference trend, by 2050, the best-case scenario (S1) suggests that halving the SEV for diets high in sodium could lead to an additional 5.2 averted DALY rate (95% PI 4.8–5.7) in the sex-combined analysis (8.3, 95% PI 7.6–9.1 for males; 2.0, 95% PI 1.8–2.1 for females) (Supplementary Table S2).

### 3.3. Age-Specific Burden Projections

**Figure 2:**
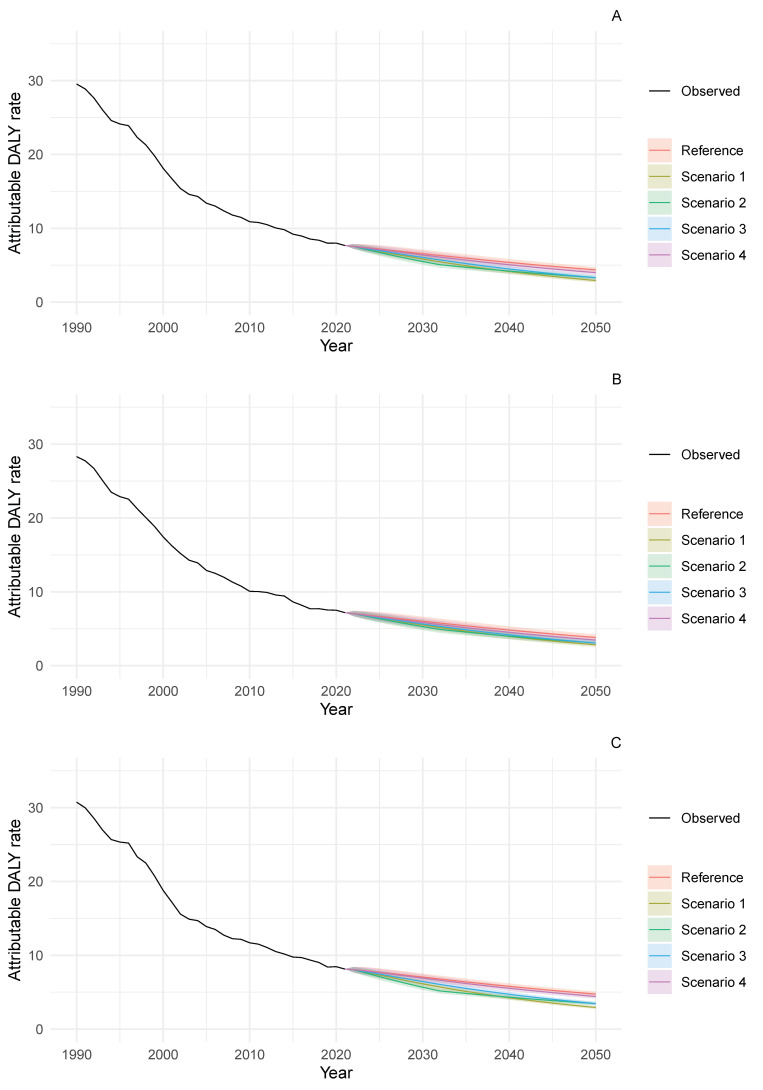
Trends in DALY rates attributable to diets high in sodium for stomach cancer among adults aged 15–49 years in Japan, 1990–2050. (**A**) Sexes combined, (**B**) females and (**C**) males. DALY rates are per 100,000 population. Lines show observed data (1990–2021) and projections (2022–2050) with 95% prediction intervals. Scenario definitions as in Figure 1.

The burden of stomach cancer among populations aged 15–49 years was substantially lower than in older age groups. In this younger age group, the projected gap between the reference and alternative scenarios in 2050 was relatively narrow (<2 DALYs per 100,000 population across all sex categories) (Figure 2). The best-case scenario (S1) forecast attributable DALY rates of 2.9 (95% PI 2.7–3.1), 2.8 (95% PI 2.5–3.1) and 2.9 (95% PI 2.7–3.2) for males, females and both sexes combined, respectively—representing substantial improvements from 2021 rates of 8.1, 7.2 and 7.7 for the same categories (Supplementary Table S3).

**Figure 3:**
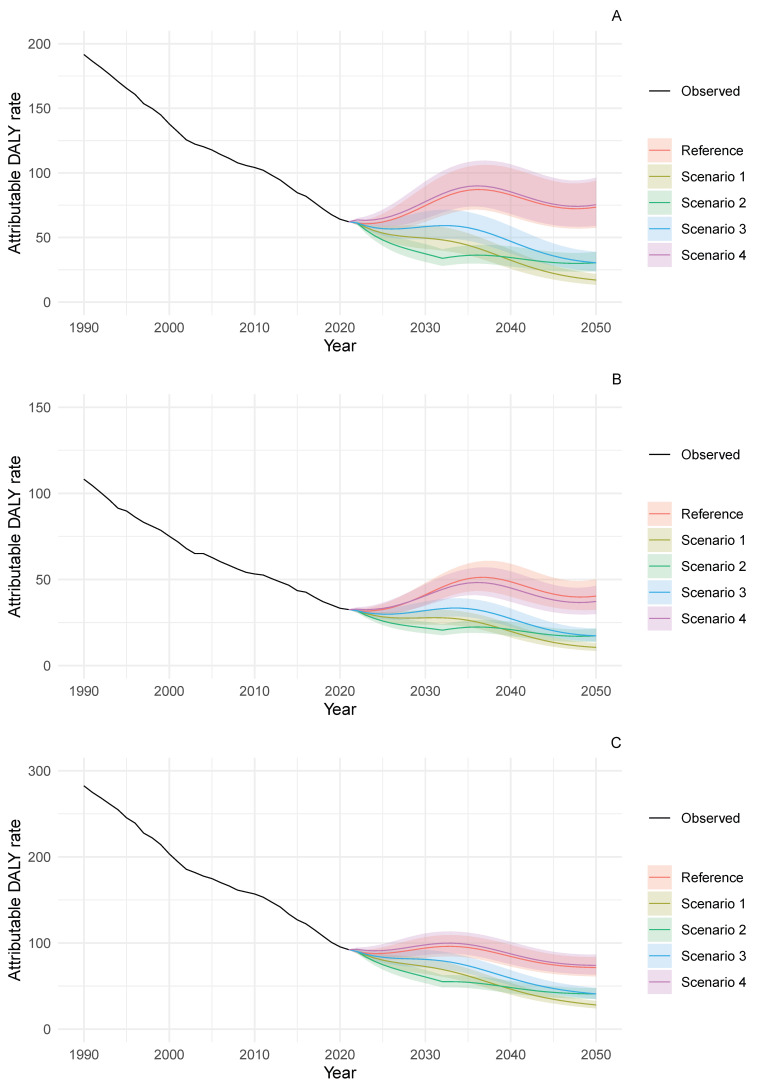
Trends in DALY rates attributable to diets high in sodium for stomach cancer among adults aged 50–69 years in Japan, 1990–2050. (**A**) Sexes combined, (**B**) females and (**C**) males. DALY rates are per 100,000 population. Lines show observed data (1990–2021) and projections (2022–2050) with 95% prediction intervals. Scenario definitions as in Figure 1.

**Figure 4:**
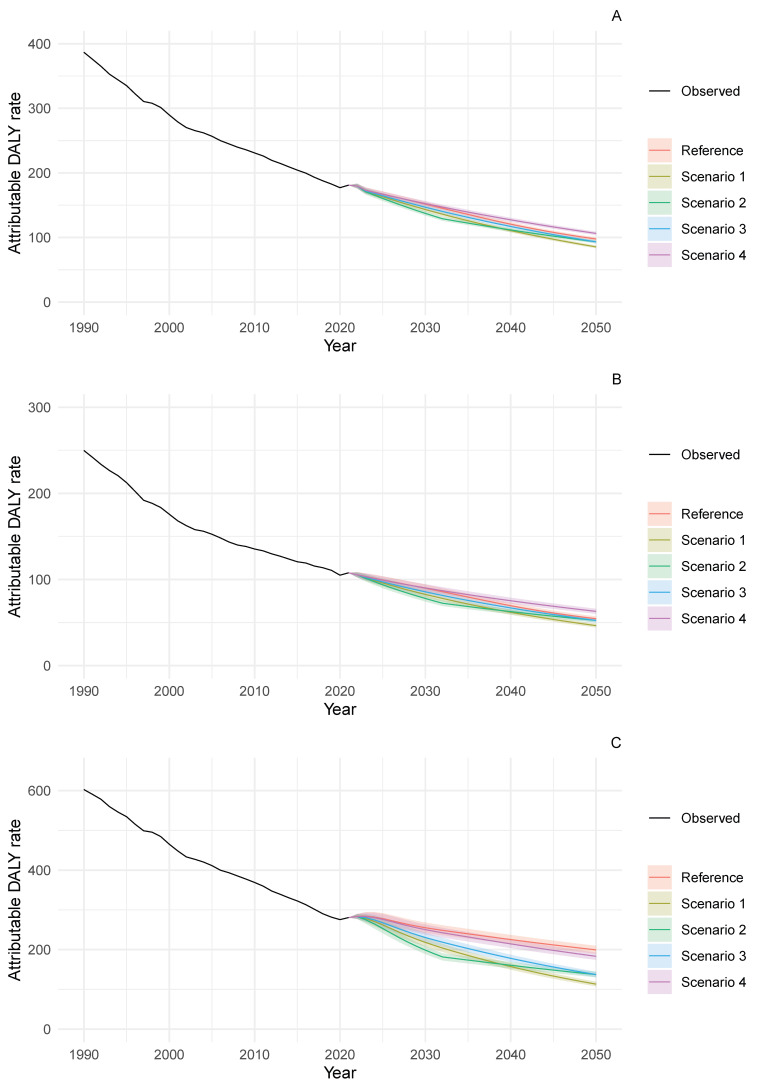
Trends in DALY rates attributable to diets high in sodium for stomach cancer among adults aged ≥ 70 years in Japan, 1990–2050. (**A**) Sexes combined, (**B**) females and (**C**) males. DALY rates are per 100,000 population. Lines show observed data (1990–2021) and projections (2022–2050) with 95% prediction intervals. Scenario definitions as in Figure 1.

The potential health impact of dietary sodium reduction, as defined by scenarios S1–S4, showed marked differences in attributable DALY trends among adults aged 50–69 years across all sex categories (Figure 3) and males aged ≥70 years (Figure 4). By 2050, the projected DALY rates between the best-case (S1) and reference scenarios in the 50–69-year-old age group differed by 30 to 56 DALY per 100,000 population depending on sex: 28.1 (95% PI 24.0–32.8) versus 71.5 (95% PI 61.2–83.6) for males; 10.6 (95% PI 8.5–13.1) versus 40.3 (95% PI 32.5–50.2) for females; and 17.1 (95% PI 13.4–21.8) versus 73.5 (95% PI 57.5–93.0) for sexes combined (Supplementary Table S4).

While the potential for burden reduction across scenarios in the ≥70-year-old age group was less pronounced than in the 50–69-year-old category, the attributable DALY rates per 100,000 population remained notably high in 2050, particularly for males under the reference scenario (199.4, 95% PI 189.4–209.6). Substantial improvements in the burden of stomach cancer attributable to diets high in sodium could still be achieved if SEV reductions reached the levels simulated in scenarios S1–S3 (Supplementary Table S5).

## 4. Discussion

Our forecasting analysis of DALYs attributable to high sodium intake for stomach cancer in Japan from 2022 to 2050 highlights several findings of relevance to national and global public health policy. Throughout, these findings are best interpreted as modelled associations between population-level dietary exposure metrics and projected DALYs rather than as direct causal estimates, and the differences between scenarios reflect counterfactual contrasts under explicit assumptions about the future trajectory of dietary sodium exposure rather than predicted real-world outcomes. Furthermore, the 95% prediction intervals around the modelled trajectories overlap substantially across adjacent policy scenarios; this overlap means that, although the point-estimate gradient by sodium reduction ambition is consistent and ordered, adjacent scenarios cannot be regarded as statistically distinct at conventional confidence levels and should be interpreted as an ordering of plausible health gains under varying levels of policy ambition rather than as differential point predictions. Most notably, our results demonstrate concerning patterns in recent dietary sodium exposure trends, with increases observed across most demographic groups during the past five years—a finding that stands in stark contrast to historical declining trends and previous forecasts [19,21,29]. These findings are corroborated by observational data from the National Health and Nutrition Survey (NHNS), an annual household survey conducted by the Ministry of Health, Labour and Welfare of Japan [25]. The survey data indicate that the shift from decline to stagnation, followed by a subsequent increase, began around 2015. The age-adjusted daily salt intake (standardised to the 2010 census population) for adults aged 20 years and older peaked in 2018, with males consuming 11.0 g/day and females 9.3 g/day—levels comparable to those observed in 2013 [25]. This reversal highlights the urgent need for renewed attention to sodium reduction strategies, particularly in Japan, a country with traditionally high sodium consumption. This dietary pattern reflects deep-rooted culinary practices, including traditional seasonings (soy sauce and miso) and fermented and preserved foods; such dietary challenges are shared across many cultures worldwide, each with their own traditional salt-preserved foods and seasonings. Japan’s ongoing efforts to gradually align with global recommendations while maintaining cultural sensitivity could therefore serve as a valuable model for similar initiatives worldwide [30].

Our findings extend the prior modelling evidence base in four concrete ways. First, to our knowledge, this is the first forecast of stomach-cancer-attributable DALYs in Japan that is explicitly benchmarked against the revised “Health Japan 21” (third term) target of 7 g/day by 2032 [22]; the closest earlier Japanese modelling work was anchored to the second-term 8 g/day target and a 2040 horizon and covered salt-attributable burden across cardiovascular disease, chronic kidney disease and stomach cancer jointly [21], while prior studies covering Japan within a five-country Asian descriptive analysis [19] or a single cross-sectional national estimate [20] did not model future policy trajectories at all. Second, we use GBD 2021, which incorporates the Burden-of-Proof framework and updated exposure–response estimates for dietary sodium [5,24], allowing re-estimation of projections under the most current evidence. Third, by incorporating the NHNS-observed stalling of salt intake reduction after 2015 [25], our reference and worst-case trajectories capture a plausibility that pre-2020 projections—which assumed continued monotonic decline—could not. Fourth, by stratifying forecasts jointly by sex and age group, our estimates quantify how much of the population-level benefit of each policy scenario is concentrated in, or missed by, particular demographic strata—information that is directly relevant to the targeting of national sodium-reduction interventions.

The variation in SEV trends across different age groups offers valuable insights for targeted interventions. While the population aged 50–69 years showed continued decreases in sodium exposure, other age groups demonstrated increasing trends. According to NHNS measured data, this age group showed a marginally detectable increasing trend around 2018, similar to other age groups [25]. While these SEV results may be attributed in part to inherent uncertainties in GBD modelling [24], the heterogeneity in exposure patterns suggests that existing sodium reduction strategies may have varying effectiveness across age groups. These differences likely reflect variations in dietary habits, food choices and responsiveness to public health messaging. This pattern is particularly evident in Japan, where analysis of dietary patterns between 2007 and 2019 revealed that younger adults (20–39 years) were less responsive to salt reduction efforts than older age groups [29]. While adults aged 40 years and above demonstrated a gradual decrease in sodium intake from seasonings—the primary source, accounting for 70% of dietary sodium in Japan—younger adults showed minimal changes in their seasoning-derived sodium intake [29].

Our scenario analyses demonstrate the effects of sodium reduction policies on future stomach cancer burden in Japan. The best-case scenario (S1), targeting a 50% reduction in SEV by 2050, showed reductions in stomach cancer DALY rates across all age groups, with particularly pronounced effects in the 50–69-year-old age group. These findings are consistent with previous research on the benefits of population-level sodium reduction strategies across diverse settings and outcomes. For example, Aminde et al. modelled that achieving WHO’s recommended 30% reduction in population-wide sodium intake in Cameroon could reduce incidence of ischaemic heart disease, stroke subtypes and hypertensive heart disease by approximately 5–13% over the lifetime and gain approximately 776,400 health-adjusted life years, with a projected 16.8% relative reduction in the probability of premature cardiovascular disease mortality between 2016 and 2030 [15]. Similar, though more modest, cardiovascular benefits have been modelled in Singapore under progressive reductions in daily salt intake and in Australia under mandatory packaged-food sodium benchmarks [16,17]. In the context of stomach cancer aetiology in Japan, it is important to acknowledge that *H. pylori* infection is the most important risk factor [31]. The present study therefore intentionally focuses on high sodium intake as an independently modifiable dietary exposure that is directly amenable to population-level policy intervention. Stomach cancer aetiology is, however, multifactorial—encompassing *H. pylori* infection, tobacco smoking, alcohol consumption, host genetic predisposition, and broader dietary patterns—and our model does not explicitly capture these other determinants. The projected DALY rates and inter-scenario differences therefore quantify only the salt-attributable component of future stomach cancer burden, and should not be interpreted as deterministic predictions of the total burden that would remain after a real-world sodium reduction policy. High salt intake and *H. pylori* infection are thought to act synergistically in promoting gastric carcinogenesis [11], suggesting that sodium reduction should be viewed as a complementary rather than competing component of a comprehensive approach to stomach cancer prevention [10,32]. The magnitude of difference between scenarios, especially between the best-case and reference scenarios, illustrates how sodium reduction policies could influence future stomach cancer burden. These quantitative estimates of health gains under various policy approaches provide valuable information for policymakers and public health agencies managing future risks and disease prevention, and emphasise the importance of investing in and implementing interventions to reduce salt intake and consumption of highly salted foods as a means of controlling cancer burden [20].

Looking beyond East Asia, high salt intake does not always translate into a proportionally high salt-attributable stomach cancer burden. Mean adult salt intakes exceeding 11 g/day in several Central and Eastern European populations—for example, Hungary (14.4 g/day), Czechia (13.0 g/day), Slovakia (12.9 g/day), Bulgaria (12.9 g/day), Romania (12.9 g/day) and Poland (11.1 g/day)—are comparable to those in South Korea (12.3 g/day) [33], yet GBD 2021 reports 2021 age-standardised salt-attributable DALY rates of 23.5 per 100,000 in Eastern Europe and 23.1 in Central Asia versus 41.1 across East Asia overall and only 8.9 in Western Europe [14]. Three classes of modifying factors plausibly explain this incomplete proportionality: *H. pylori* prevalence and CagA strain composition (central to the salt–*H. pylori* synergy); the dietary matrix delivering sodium (processed packaged foods vs. fermented salt-preserved traditional foods); and population-level genetic susceptibility, including East Asian polymorphisms in IL1B and TNF. Modelled effect sizes derived from East Asian data should therefore not be transposed unmodified to settings with markedly different risk factor profiles.

The observed patterns in attributable stomach cancer DALY rates across age groups and scenarios carry important implications for health equity and resource allocation. The substantial burden among older age groups, particularly males aged ≥ 70 years, suggests that targeted interventions for these populations could yield significant health benefits. However, the impact of high salt intake on stomach cancer risk is not limited to older populations. A dose–response relationship between salt intake and stomach cancer risk has been observed across age groups [10,34]. These findings highlight the importance of life-course approaches to sodium reduction, as the cumulative effects of long-term high salt consumption can significantly increase cancer risk over time. The persistence of high attributable DALY rates among adults aged ≥ 70 years across all sodium reduction scenarios is best understood within a life-course epidemiology framework. By age 70, several decades of high salt exposure—often combined with historical *H. pylori* colonisation—may already have affected the gastric mucosa through mechanisms, including mucosal barrier damage, inflammation and enhanced *H. pylori*-related gastric carcinogenesis [10]. A substantial proportion of salt- and *H. pylori*-related gastric mucosal damage may therefore have accumulated before any late-life sodium reduction. Sodium reduction in adults aged ≥70 years can still confer meaningful absolute reductions, particularly given the very high baseline DALY rate, but the marginal benefit per unit of exposure reduction is expected to be smaller than for younger adults with shorter cumulative exposure histories. This argues for prioritising sodium reduction interventions beginning in childhood, adolescence and early adulthood while preserving secondary prevention, including endoscopic screening and *H. pylori* test-and-treat strategies, for older adults in whom primary-prevention gains are likely to be more limited.

Our findings also contribute to the broader global discourse on sodium reduction targets and strategies. While Japan’s national health promotion policy “Health Japan 21” (third term) sets more ambitious targets than previous iterations, our analysis suggests that even achieving these targets may be insufficient to meet the WHO’s global recommendations. This gap between national and international targets is not unique to Japan and reflects a common challenge faced by many countries in balancing ambitious public health goals with practical implementation constraints [35]. Our scenario analyses of potential health impacts at various target levels can provide insights for policy discussions in other high-sodium-consuming nations, helping countries understand the potential benefits of more ambitious targets and informing the development of comprehensive sodium reduction strategies.

From a translational perspective, the policy scenario contrasts modelled here can be operationalised in Japan through three complementary levers, mapped to the WHO sodium reduction “Best Buys”. First, food reformulation—the most scalable structural intervention—is now coordinated through the Ministry of Health, Labour and Welfare’s Strategic Initiative for a Healthy and Sustainable Food Environment, with industry-targeted salt reduction guidance [36]; international experience suggests that voluntary reformulation can plateau, and a Japan-focused economic simulation found that mandatory reformulation under a best-case cost scenario might be economically preferable to other modelled sodium reduction policies [37]. Second, consumer-facing measures—front-of-pack labelling, mass-media campaigns aligned with Health Japan 21 (third term), and dietary education through schools and workplaces—can shift demand and amplify supply-side reformulation [38]. Third, regulatory and procurement levers (sodium ceilings for institutional catering and public procurement criteria favouring low-sodium products) further support population-level shifts. Sodium reduction policies are best implemented in parallel with *H. pylori* test-and-treat strategies, tobacco control and continued endoscopic screening, so that the salt-attributable health gains projected here are realised as part of an integrated stomach cancer prevention and control programme [35].

### Limitations

Several important limitations should be considered. First, as with any forecasting study, our predictions are inherently subject to uncertainty and are based on the assumption that observed patterns and relationships will continue into the future [39]. While short-term dependency was controlled in the ARIMAX model, the recent increase in sodium exposure trends demonstrates the challenges in long-term forecasting and emphasises the importance of regular monitoring and model updating. Further research is needed to better understand the drivers of these recent trends, particularly the factors contributing to increased sodium exposure among certain age groups. It should be noted that, owing to the COVID-19 pandemic, the NHNS was not conducted in 2020 and 2021. The most recent survey in 2023 shows that the age-adjusted daily salt intake for adults aged 20 years and older decreased to 10.6 g/day for males and 8.9 g/day for females compared with 2018, but these values are slightly higher (0.1 g/day for both sexes) than those observed in 2022, making it difficult to definitively confirm a return to the historical declining trend [25].

Second, although the SEV serves as a useful proxy for population-level sodium exposure, it may not fully capture all aspects of dietary sodium intake or account for individual-level variations. The relationship between SEV and actual sodium intake, while established, may be influenced by various factors not captured in our model, including changes in food processing technologies, shifts in dietary patterns and evolving food policies. Because SEV is a modelled composite metric derived from multiple underlying data sources within the GBD framework, it is not directly equivalent to biomarker-validated individual-level dietary assessments. Although 24 h urinary sodium excretion is widely regarded as a gold-standard measure in nutritional epidemiology, such individual-level biomarker data were not available as direct input in the present analysis, and the extent of any resulting measurement error or attenuation bias cannot be formally quantified. Moreover, because SEV is expressed as a dimensionless, risk-weighted exposure metric rather than an absolute intake measure, our findings cannot be translated directly into grams-per-day terms comparable with the Dietary Reference Intakes for Japanese, which limits the immediate quantitative specificity of our policy implications. In addition, because the dietary composition of high-sodium food sources in Japan, including miso, traditional fermented foods and soy sauce, differs substantially from that of other high-sodium-consuming countries, the SEV-based exposure patterns observed here may not be directly generalisable to other national contexts, and caution is warranted when drawing international comparisons. More specifically, the external validity and generalisability of the present projections may be limited by differences in the sources and dietary context of sodium intake across countries. In Japan and East Asia, high salt exposure is closely linked to traditional dietary patterns, including pickled foods, soy sauce and miso, whereas the composition of high-sodium diets and the accompanying risk factor profile may differ substantially in other settings [14]. Consequently, the absolute magnitudes and policy impact estimates derived here for Japan should not be extrapolated unmodified to other national settings; equivalent country-specific modelling incorporating local dietary patterns and risk factor structures would be required before the present projections could be applied to a non-Japanese context.

Third, our model focuses specifically on the burden of stomach cancer attributable to high sodium intake and does not account for potential interactions with other risk factors, such as *H. pylori* infection, genetic susceptibility or other dietary factors. This focused approach may not capture the full complexity of stomach cancer aetiology or the comprehensive health impacts of sodium reduction interventions. The deliberate restriction of scope to stomach cancer reflects the well-established mechanistic and epidemiological evidence linking dietary sodium to gastric carcinogenesis and the primary research objective of the present study. It should nonetheless be acknowledged that high sodium intake is also a recognised risk factor for other major health outcomes, including hypertension, cardiovascular disease and oesophageal cancer [40,41,42]; the burden attributable to these outcomes was not modelled, and a multi-outcome analysis remains an important direction for future research.

Fourth, while our scenarios provide valuable insights into potential policy impacts, they represent simplified trajectories and may not fully account for the complex social, economic and cultural factors that influence dietary behaviours and policy implementation. These factors are accordingly assumed to remain constant across the study period. The feasibility of achieving the modelled reductions in sodium exposure would depend on multiple factors, including political will, industry cooperation and public acceptance of interventions. Relatedly, the reference and worst-case trajectories rest on the assumption that historical exposure–outcome relationships will continue into the future. The post-2015 stalling of progress in Japanese population salt-intake reduction, together with potential future shifts in food supply structure, packaged-food consumption and consumer dietary preferences, indicates that this continuity-of-trend assumption may not hold over the 2022–2050 horizon. The reference scenario should therefore be read as one plausible continuation of recent trends rather than as a most likely future, and the inter-scenario differences are best understood as plausible directional bounds under each set of explicit exposure assumptions.

Fifth, readers should exercise caution when interpreting inter-scenario comparisons. Several projected differences in DALY rates between scenarios, particularly between the reference and worst-case scenarios, or between the two 30–reduction scenarios, are modest in absolute terms and may overlap with the 95% prediction intervals of adjacent scenarios. These comparisons are therefore best understood as indicative of directional trends and plausible magnitudes of effect under different policy conditions rather than as precise or statistically definitive point estimates.

Sixth, the robustness of the ARIMAX framework merits explicit consideration. ARIMAX models assume linearity in the relationship between log-DALY rates and the predictor variables (SEV and SDI), stationarity of the differenced series, and structural stability of the underlying data-generating process across the 1990–2050 projection horizon. The assumptions related to stationarity were assessed using standard diagnostic procedures, including the augmented Dickey–Fuller test for stationarity, the Ljung–Box test for residual autocorrelation, and visual inspection of standardised residuals. Models were retained for forecasting when these diagnostics indicated no appreciable departure from the underlying ARIMAX assumptions. By contrast, structural stability of the data-generating process cannot be examined from the historical series alone. Sustained departures in dietary behaviour, sodium exposure, or *H. pylori* prevalence from their 1990–2021 trajectories could, in principle, affect the validity of long-horizon extrapolation. The post-2015 stalling of progress in Japanese population salt intake reduction, as reported by the National Health and Nutrition Survey, illustrates that such departures are not merely hypothetical. Long-horizon ARIMAX projections to 2050 should therefore be interpreted as conditional extrapolations under explicit exposure scenarios.

## 5. Conclusions

This study presents a scenario-based forecast of stomach cancer burden attributable to high sodium intake in Japan through 2050. Under modelled assumptions, sustained achievement of national sodium reduction targets could meaningfully reduce future stomach cancer DALYs relative to a continuation of recent trends, with the largest absolute gains in older adults but the largest relative gains in younger and middle-aged groups. Because stomach cancer aetiology is multifactorial and projections rest on modelled associations and a continuity-of-trend assumption, our findings support strengthened, demographically targeted sodium reduction interventions as one complementary component of a broader, multi-risk-factor approach to stomach cancer prevention in Japan, and underscore the importance of regular monitoring and evidence-based policy adjustment.

## Figures and Tables

**Table 1:** Age-standardised DALY rates attributable to diets high in sodium for stomach cancer and population characteristics, Japan, 1990–2021.

Year	Sex	Attributable DALY Rate Per 100,000	SEV (Diets High in Sodium)	SDI
1990	Both	65.73	59.38	0.79
	Male	93.97	64.95	
	Female	43.97	53.92	
1995	Both	55.61	58.66	0.81
	Male	80.69	64.67	
	Female	36.16	52.73	
2000	Both	46.55	53.06	0.82
	Male	68.23	59.11	
	Female	29.47	47.08	
2005	Both	38.86	45.72	0.83
	Male	57.55	52.08	
	Female	23.79	39.42	
2010	Both	32.93	41.25	0.84
	Male	49.45	48.20	
	Female	19.29	34.36	
2015	Both	27.10	39.24	0.86
	Male	40.43	45.73	
	Female	15.93	32.81	
2021	Both	22.26	39.60	0.87
	Male	33.08	45.58	
	Female	13.04	33.63	

Note: DALY rates are expressed per 100,000 population. SEV = summary exposure value; SDI = socio-demographic index. While selected years are presented for clarity, all analyses utilised complete yearly data.

## Data Availability

All data analysed in this study are publicly available. Age-, sex- and year-specific DALY rates for stomach cancer attributable to diets high in sodium, SEV values and SDI values were obtained from the Global Burden of Disease Results tool (https://vizhub.healthdata.org/gbd-results/, accessed on 23 January 2025) maintained by the Institute for Health Metrics and Evaluation. No new primary data were generated for this study. Analytical code is available from the corresponding author upon reasonable request.

## Supplementary Materials

The following supporting information can be downloaded at: https://www.mdpi.com/article/10.3390/nu18101641/s1, Table S1: DALY rates attributable to diets high in sodium for stomach cancer and population characteristics by age group, 1990–2021. (A) Ages 15–49 years, (B) ages 50–69 years, and (C) age ≥70 years; Table S2: Projected age-standardised DALY rates attributable to diets high in sodium for stomach cancer, 2022–2050. (A) Sex combined, (B) females, and (C) males; Table S3: Projected DALY rates attributable to diets high in sodium for stomach cancer among population aged 15–49 years, 2022–2050. (A) Sex combined, (B) females, and (C) males; Table S4: Projected DALY rates attributable to diets high in sodium for stomach cancer among population aged 50–69 years, 2022–2050. (A) Sex combined, (B) females, and (C) males; Table S5: Projected DALY rates attributable to diets high in sodium for stomach cancer among population aged ≥70 years, 2022–2050. (A) Sex combined, (B) females, and (C) males; Figure S1: Trends in summary exposure value for diets high in sodium by sex and age groups in Japan, 1990–2021. (A) Ages 15–49 years, (B) ages 50–69 years, (C) and age ≥70 years (all panels in Figure S1 were generated by the authors from publicly available Global Burden of Disease 2021 estimates and are not reproduced from the literature); Figure S2: Association between daily salt intake and summary exposure value for diets high in sodium in Japan, 1990–2016.
