# Supplementary material for: Forecasting Stomach Cancer Burden from High Sodium Intake in Japan, 2022–2050: Scenario Analysis of Demographic Disparities
Source: Nutrients. 2026 May 21;18(10):1641. doi: 10.3390/nu18101641 (PMC13209551; doi:10.3390/nu18101641)
Supplement: Supplementary file 1 [file nutrients-18-01641-s001.zip › Supplementary figures.pdf]

Supplementary Figure S1. Trends in summary exposure value for diets high in sodium by sex and age groups in Japan, 1990-2021. (A) Ages 15-49 years, (B) Ages 50-69 years, (C) Ages ≥70 years. *Source: Global Burden of Disease 2021 Results (Institute for Health Metrics and Evaluation), processed and plotted by the authors.*

Panel A. Ages 15-49 years

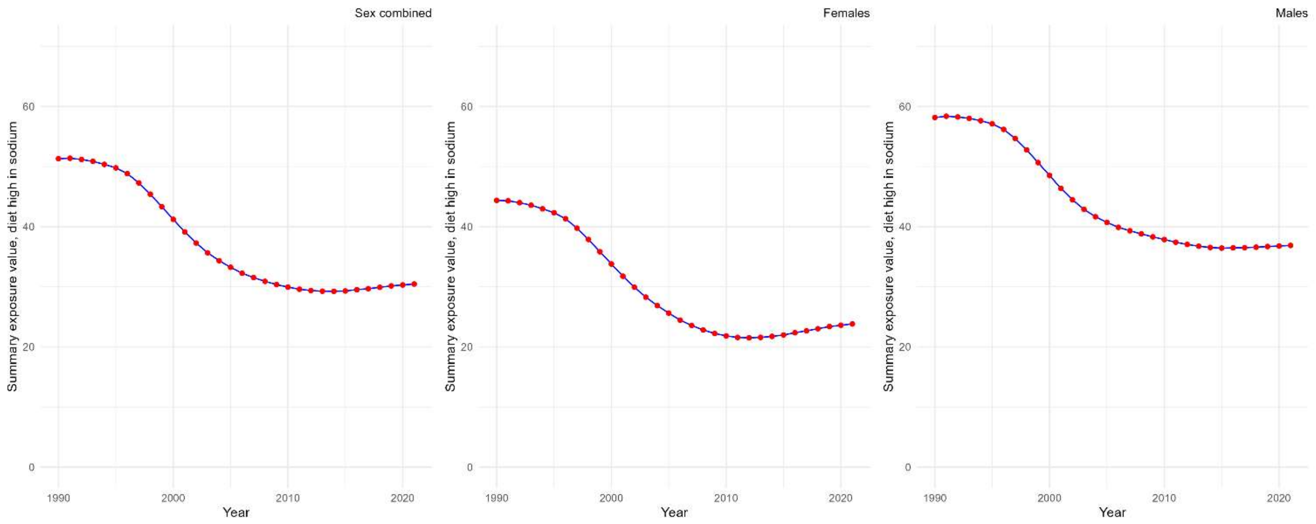

Panel B. Ages 50-69 years

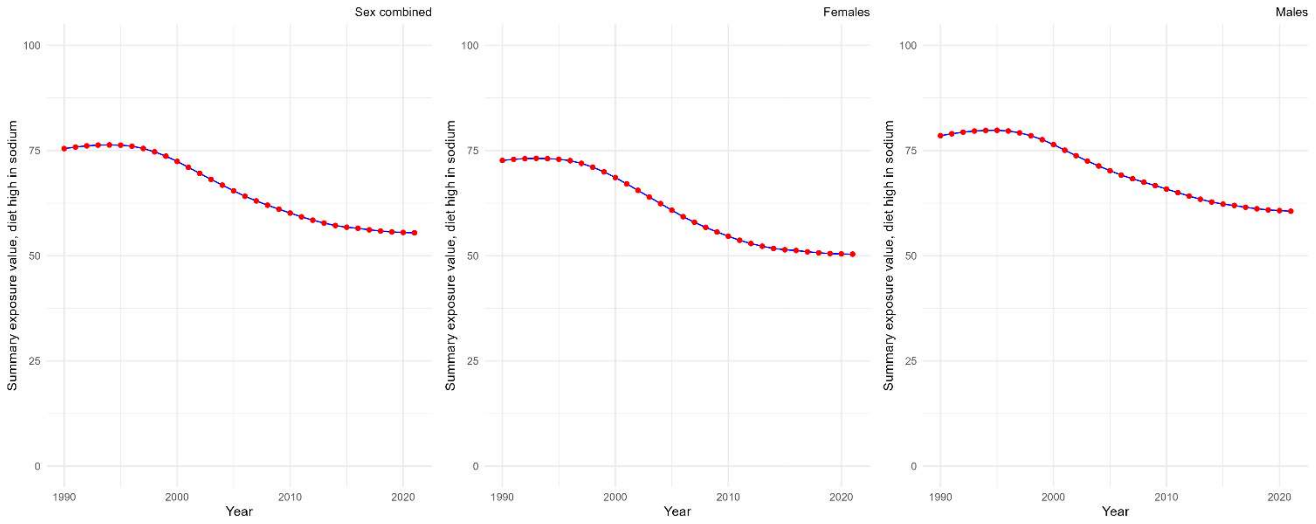

Panel C. Ages ≥70 years

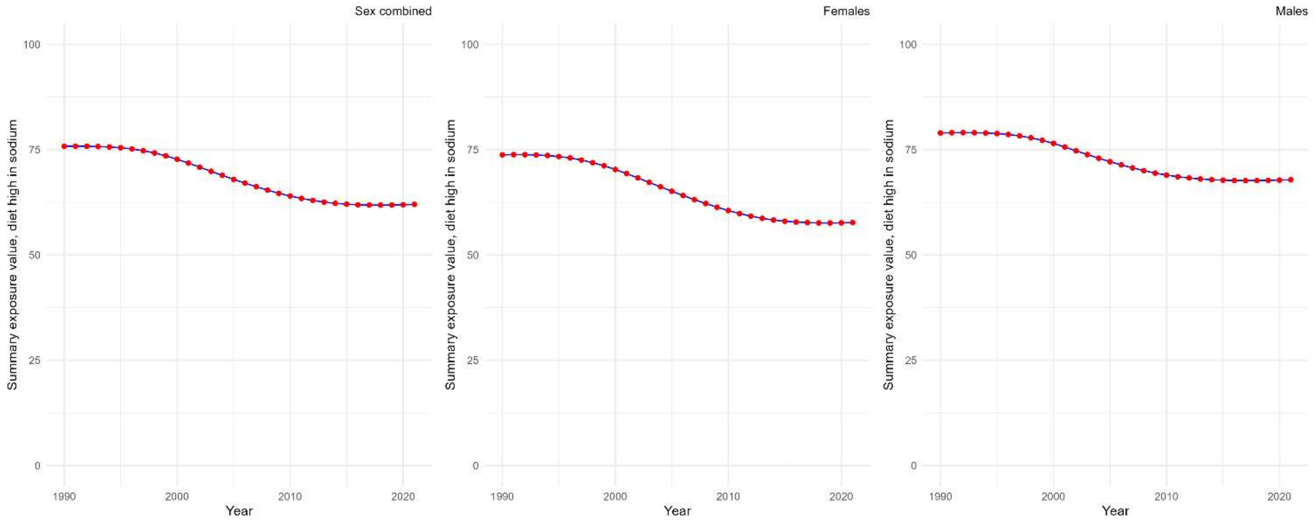

Supplementary Figure S2. Association between daily salt intake and summary exposure value for diets high in sodium in Japan, 1990-2016.

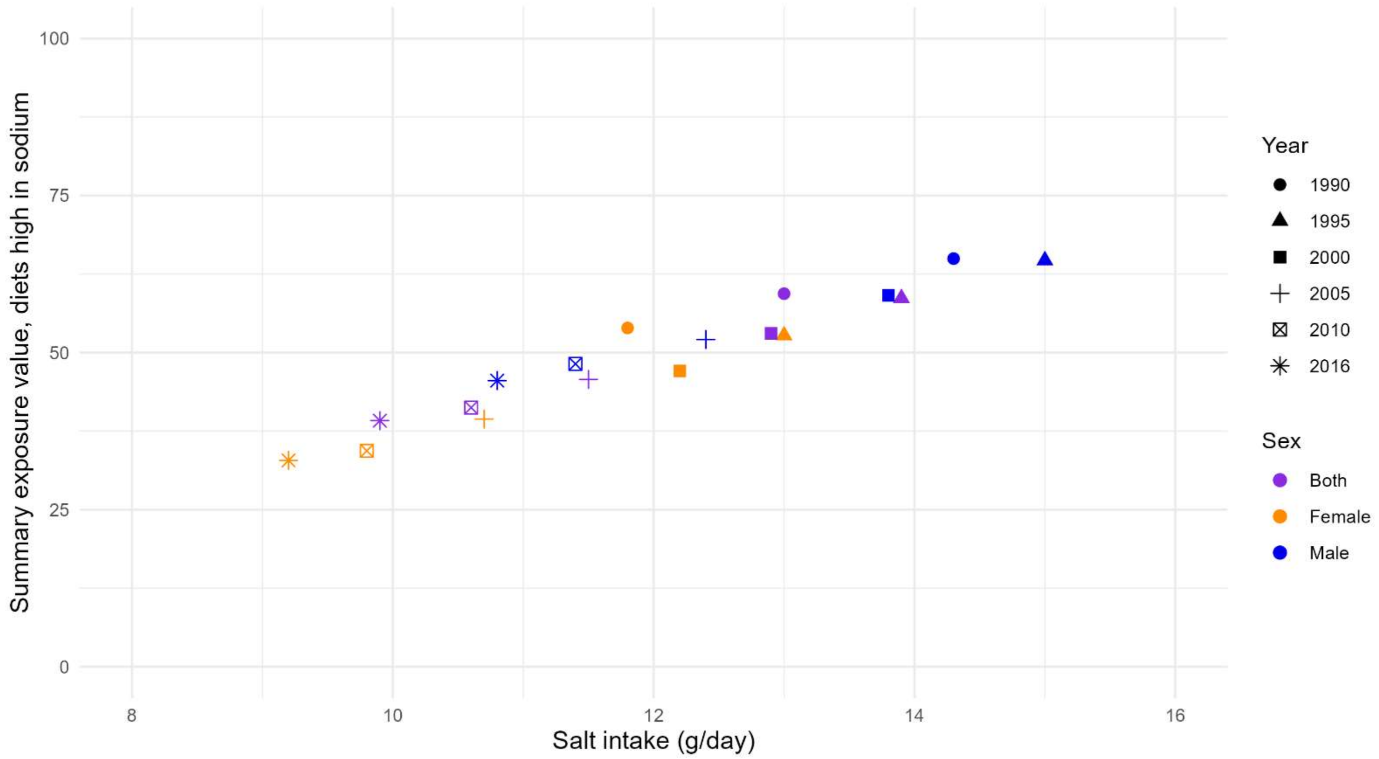

Source: Salt intake data from Nomura et al (2020).[21]

Summary exposure value for diet high in sodium from GBD Results (IHME).[26]
